# The Effect of Biochars and Endophytic Bacteria on Growth and Root Rot Disease Incidence of *Fusarium* Infested Narrow-Leafed Lupin (*Lupinus angustifolius* L.)

**DOI:** 10.3390/microorganisms8040496

**Published:** 2020-03-31

**Authors:** Dilfuza Egamberdieva, Vyacheslav Shurigin, Burak Alaylar, Hua Ma, Marina E. H. Müller, Stephan Wirth, Moritz Reckling, Sonoko Dorothea Bellingrath-Kimura

**Affiliations:** 1 Leibniz Centre for Agricultural Landscape Research (ZALF), 15374 Müncheberg, Germany; Hua.Ma@zalf.de (H.M.); mmueller@zalf.de (M.E.H.M.); swirth@zalf.de (S.W.); Moritz.Reckling@zalf.de (M.R.); Sonoko.Bellingrath-Kimura@zalf.de (S.D.B.-K.); 2 Faculty of Biology, National University of Uzbekistan, Tashkent 100174, Uzbekistan; slaventus87@inbox.ru; 3 Faculty of Arts and Sciences, Department of Molecular Biology and Genetics, Agri Ibrahim Cecen University, 04100 Agri, Turkey; balaylar@agri.edu.tr; 4 Faculty of Life Science, Humboldt University of Berlin, 14195 Berlin, Germany

**Keywords:** biochar, root rot, lupin, Fusarium, biological control, legumes

## Abstract

The effects of biochar on plant growth vary depending on the applied biochar type, study site environmental conditions, microbial species, and plant–microbial interactions. The objectives of the present study were therefore to assess 1) the response of growth parameters of lupin and root disease incidence to the application of three biochar types in a loamy sandy soil, and 2) the role of endophytic bacteria in biological control of root rot disease incidence in lupin after the amendment of soil with different biochar types. As biochar types we tested (i) hydrochar (HTC) from maize silage, (ii) pyrolysis char from maize (MBC), and (iii) pyrolysis char from wood (WBC) at three different concentrations (1%, 2%, and 3% of char as soil amendments). There were no significant effects in lupin shoot and root growth in soils amended with WBC at any of the concentrations. MBC did not affect plant growth except for root dry weight at 2% MBC. HTC char at 2% concentration, significantly increased the root dry weight of lupin by 54–75%, and shoot dry weight by 21–25%. Lupin plants grown in soil amended with 2% and 3% WBC and MBC chars showed 40–50% and 10–20% disease symptoms, respectively. Plants grown in soil without biochar and with HTC char were healthy, and no disease incidence occurred. *Pseudomonas putida* L2 and *Stenotrophomonas pavanii* L8 isolates demonstrated a disease reduction compared to un-inoculated plants under MBC and WBC amended soil that was infested with *Fusarium solani*.

## 1. Introduction

Biochar is a solid by-product of biomass produced by pyrolysis or by high temperatures under limited supply of or in the complete absence of air [1]. It is considered to be a tool for carbon sequestration and to improve soil and crop health [2,3,4]. The amendment of soil with biochar was reported to enhance plant growth and yields, such as of pepper and tomato [5], soybean [6], maize [7], or wheat [8]. Moreover, biochar holds the potential as a suitable carrier of microbial inoculants to improve plant growth [9]. The biochar application improved soil cation exchange capacity [10], water holding capacity [11], and soil organic matter [12]. In addition, biochar application was found to reduce plant diseases by inducing systemic resistance in plants against different fungal pathogens [13], including *Fusarium oxysporum* f. sp. *lycopersici* on tomato [14], *Botrytis cinerea* on tomato [15], and *Rhizoctonia solani* on cucumber [16]. It has also been reported that biochar application into soil improved soil and plant microbiomes, especially beneficial microbes that can directly improve plant fitness [6,17,18]. The roles of endophytes in plant health, especially to protect their host plants against invasion and damage by soil-borne pathogens have been reported in numerous studies [19,20,21,22]. For example, root rotting of mung bean caused by *Fusarium solani* was reduced by endophytic bacteria *Pseudomonas* spp. isolates from root nodules [23].

A combined application of biochar and endophytic bacteria into the soil could provide more benefits compared to the single inoculation of plants with bacteria [9]. This was explained by the favorable conditions provided by biochar for bacterial survival and activity. However, there is increased evidence that the responses of plant growth, nutrient acquisition, disease incidence, soil biogeochemical processes, or microbial communities after biochar application depend on the type of feedstock and the thermal conditions during production (e.g., heating period and the final set point temperature) [24]. For example, Butnan et al. [25] observed that plant growth was reduced in a sandy Ultisol amended with eucalyptus wood-derived biochar produced by high (800 °C) temperatures, whereas biochar produced at 350 °C enhanced plant growth. It has also been noted that biochar types have an impact on plant susceptibility to pathogens, and it may enhance plant disease incidence and severity (reviewed by Frenkel et al. [26]). However, more detailed knowledge is needed to improve our understanding of the functional relationships operating behind the effects of different biochar types on plant disease incidence and the mechanisms used by bacteria to boost plant fitness.

Narrow-leafed lupin (*Lupinus angustifolius* L.) is a common grain legume crop in Europe, the Mediterranean countries [27], and Australia. Lupin forms nitrogen-fixing nodules that support plants to meet their N needs through which plant biomass and yield increase [28]. The fixed nitrogen is also available to subsequent crops, reducing the requirement for nitrogen fertilizers, and crops grown in rotation with lupin benefit from positive rotational effects [29]. Greater production of lupin and other grain legumes would benefit many ecosystem services [30]. However, lupin is susceptible to soil-borne pathogens such as *F. oxysporum* and *F. solani* [31] reducing plant growth rates, plant nutrition, and thus resulting in yield losses and relatively low yield stability [32].

Based on a pot experiment, our objectives were to assess 1) the response of growth parameters of lupin and disease incident to the application of three types of biochar applied in a loamy sandy soil, and 2) the role of endophytic bacteria in biological control of root rot disease incidence in lupin after amendment with biochar.

## 2. Materials and Methods

### 2.1. Plant, Biochar, and Soil

Seeds of the narrow-leafed lupin (*L. angustifolius* L.) cultivar Probor were obtained from the Fehrower Agrarbetrieb GmbH, Germany. We selected biochars produced from maize by two different pyrolysis techniques, i.e., heating at 600 °C (MBC) and batch-wise hydrothermal carbonization at 210 °C (HTC), and also a char derived from wood by pyrolysis at 850 °C (WBC), and they were amended to the loamy sandy soil. The chemical composition of the biochars is presented in Table 1 as characterized by Reibe et al. [33]. The biochar types were obtained from the Leibniz-Institute for Agrartechnik Potsdam-Bornim e.V. (ATB), Germany.

The soil used for the pot experiment was collected from an experimental field at the Leibniz Center for Agricultural Landscape Research (ZALF), Müncheberg, Germany. The soil consisted of clay and fine silt (7%), coarse and medium silt (19%), and sand (74%) and was characterized by the following properties: pH 6.2; organic C content 0.55%, total N content 0.07%, P content 0.03%, K content 1.25%, and Mg content 0.18% [34].

### 2.2. Plant Growth Experiment

The pot experiment was conducted under greenhouse conditions at the Leibniz Center for Agricultural Landscape Research (ZALF), Germany. Three different biochar types, WBC, MBC, and HTC, were used as soil amendments. Pots (d = 0.16 m, v = 3016 cm^3^) were filled with 1000 g of air-dried soil mixed with crushed char (particle size <3 mm) at 1%, 2%, and 3% (w/v) concentration. The following four treatments were set up:

i) plants grown in soil without WBC, MBC, or HTC biochar,
ii) plants grown in soil amended with WBC,
iii) plants grown in soil amended with MBC,
iv) plants grown in soil amended with HTC.

Each treatment included four replicated pots, arranged in a randomized complete block design with three replications (total of 12 plants), and three char concentrations (1%, 2%, and 3% w/v). Plants were grown for 30 days at a temperature of 24 °C/16 °C (day/night) and humidity of 50–60%. At harvest, the lupin root system was carefully removed, soil adhering to the roots was washed and examined for root rot symptoms. The dry weights of roots and shoots of healthy plants were determined.

### 2.3. Isolation of Fungi from Diseased Plants

The plants grown under biochar amendment (WBC) showing *Fusarium* root rot symptoms were collected, surface disinfected by dipping into 70% ethanol and then 1% NaOCl for 5 min, and were washed with sterile distilled water three times. The sterilized plants were cut into small pieces and placed on the surface of potato dextrose agar (PDA) (BD, Difco Laboratories, Detroit, USA) supplemented with chloramphenicol (Oxoid, UK) (150 ppm). The plates were kept in an incubator for five days, then fungal colonies with different morphological characteristics were isolated, and purified. The identification of fungi at the genus level was carried out by using macroscopic and microscopic examinations depending on the colony color, shape, hyphae, conidia, conidiophores, and arrangement of spores. The isolated fungal isolates belong to *Mucor* spp., *Alternaria* spp., and *Fusarium* spp.

The plant pathogenicity tests were conducted with two *Fusarium* spp. and *Alternaria* spp. isolates in order to select pathogenic fungi that cause lupin root rot disease. A single culture was prepared from each isolate. The pathogenicity of these isolates was tested by sowing lupin seeds in pots filled with sterile vermiculite and sand (1/1) mixed with spores of the pathogen (7.0 × 10^5^ spores/kg soil) and grown for six weeks. At harvest, the diseased plants were selected, and a piece of the root system was placed on a PDA plate and re-isolated. The fungal isolates were grown on sterile filter paper placed on PDA agar and then ground in liquid nitrogen. The Nucleon Phytopure kit (Amersham Biosciences GmbH, Freiburg, Germany) was used to isolate DNA. The fragments of mtSSU rDNA were amplified using MS1 and MS2 primers [35], and the sequences of the fragments were compared with those in the GenBank using the BLAST algorithm. The isolate showed 99.68% similarity with the mtSSU rDNA sequence from *F. solani* f. sp. *glycines* 91-10-1-G (GenBank Acc.N. AF125009.1) and was identified as *F. solani*.

### 2.4. Isolation of Endophytic Bacteria from Lupin Roots and Characterization

Lupin grown under field conditions at the Experimental Station of the Leibniz Center for Agricultural Landscape Research (ZALF), Germany was collected for the isolation of endophytic bacteria. Four fresh plants were separated from the shoots and roots and washed, and then surface sterilized by immersion in 70% ethanol and 5% sodium hypochlorite.

One gram of root was macerated in a mortar and mixed with 9 mL sterile buffered saline (PBS) (20 mM sodium phosphate, 150 mM NaCl, pH 7.4). The supernatant was diluted (10^1^–10^5^) and spread on Tryptic Soy Agar (TSA) (BD, Difco Laboratories, Detroit, USA). The plates were incubated for four days at 28 °C, and morphologically different colonies were picked up from the plates and purified.

#### 2.4.1. DNA Isolation

For DNA extraction, the heat treatment method [36] was used. The small aliquots of the colonies were transferred into 2-mL Eppendorf tubes with 1.5 mL of sterile MQ-water and were mixed with a Biosan B-1 Vortex for 10 sec. The tubes were incubated at 90 °C for 20 min in a Dry Block Heater (IKA Works) and centrifuged at 12,000 rpm for 5 min. The DNA-containing supernatant was taken and stored at −20 °C. The presence of DNA was checked by horizontal gel electrophoresis (0.8% agarose) and was quantified using NanoDrop™ One (Thermo Fisher Scientific, Inc. Waltham, MA).

#### 2.4.2. Polymerase Chain Reaction (PCR)

Extracted DNA was used as a template for 16S rRNA gene analysis. The 16S rRNA genes were amplified via PCR using the following primers: 16SF 5′-GAGTTTGATCCTGGCTCAG-3′ (Sigma-Aldrich, St. Louis, MO) and 16SR 5′-GAAAGGAGGTGATCCAGCC (Sigma-Aldrich, St. Louis, MO). Each 25 µL of reaction mixture contained 1 µL with 15–40 ng of DNA, 5× One Taq standard reaction buffer (BioLabs, New England)—5 µL; 10 mM dNTP mix (Thermo Scientific)—5 µL, 10 mM primer 16SF (Merck)—0.5 µL, 10 mM primer 16SR (Merck)—0.5 µL (25 pmol/mL), 0.1% bovine serum albumin (TaKaRa Bio Inc.)—1 µL, One Taq polymerase (BioLabs, New England)—0.125 µL, and MQ water—16.375 µL. The PCR was performed using a PTC-200 thermocycler (BioRad). The PCR program was as follows: a primary heating step for 30 s at 94 °C, followed by 30 cycles of denaturation for 15 s at 94 °C, annealing for 30 s at 55 °C, and extension for 1.5 min at 68 °C, followed by the final step for 20 min at 68 °C. The PCR-amplified products were examined by electrophoresis in a 0.8% agarose gel containing GelRed.

#### 2.4.3. Restriction Fragment Length Polymorphism (RFLP) Analysis

To determine the difference of the isolates similar in their color, shape, and size, we conducted an RFLP analysis of 16S rRNA products as described by Jinneman et al. [37]. The fragments of digested RNA were checked via gel electrophoresis (1% agarose gel). After the electrophoresis, the gel was visualized using a digital gel imaging system (Gel-Doc XR TM+, Bio-Rad) to identify identical isolates to reduce the number of strains to be sequenced.

#### 2.4.4. Sequencing and Phylogenetic Analysis

Before being sequenced, the PCR products were purified with the USB^®^ ExoSAP-IT^®^ PCR Product Cleanup Kit (Affymetrix, USB^®^ Products, USA) according to the manufacturer’s protocol. Sequencing was performed using an ABI PRISM BigDye 3.1 Terminator Cycle Sequencing Ready Reaction Kit (Applied Biosystems) by the manufacturer’s protocol. Received data were analyzed and corrected using Chromas (v. 2.6.5) software. Corrected sequences were merged manually using EMBOSS Explorer (http://emboss.bioinformatics.nl/). The sequences were identified using the basic local alignment search tool (BLAST), and comparisons were made with the GenBank nucleotide data bank from the National Center for Biotechnology Information (NCBI) (http://www.ncbi.nlm.nih.gov/). All sequences were multiply aligned using Clustal× 2.1 software, and a FASTA format file was used to construct the phylogenetic tree. The evolutionary history was inferred using the neighbor-joining method [38]. The optimal tree with the sum of branch length = 0.76327423 is shown. The percentage of replicate trees in which the associated taxa clustered together in the bootstrap test (500 replicates) are shown next to the branches [39]. The tree is drawn to scale, with branch lengths in the same units as those of the evolutionary distances used to infer the phylogenetic tree. The evolutionary distances were computed using the maximum composite likelihood method [40] and are in the units of the number of base substitutions per site. The analysis involved 19 nucleotide sequences. All positions containing gaps and missing data were eliminated. There were a total of 1281 positions in the final dataset. Evolutionary analyses were conducted in MEGA6 [41].

The 16S rRNA sequences isolated from the endophytic bacteria of white lupin were deposited into GenBank under the accession numbers: MH636785–MH636793.

### 2.5. Plant Growth Traits

Several plant growth-promoting traits of endophytic bacteria were determined. Indole 3-acetic acid (IAA) production was studied following the method described in Bano and Musarrat [42]. The production of chitinase and protease activity was determined as described in Malleswari and Bagyanarayan [43]. The cellulose-degrading capacity of the bacterial isolates was analyzed as described by Gupta et al. [44]. The antagonistic capabilities of bacterial isolates against the pathogenic fungi *F. oxysporum*, *F. solani*, *Fusarium culmorum*, *Verticillum dahlia*, and *R. solani* were evaluated on potato dextrose agar (PDA) media as described by Egamberdieva et al. [6].

### 2.6. Biological Control of Lupin Root Rot

The biological control of lupin root rot caused by *F. solani* under different biochar soil amendments using selected endophytes was determined in a pot experiment. For soil infestation, a suspension of *F. solani* grown in Czapek–Dox medium for four days was filtrated with sterile glass wool, centrifuged, and diluted to 10^6^ spores mL^−1^. The resulting suspension with fungal spores was mixed with soil (10^6^ spores kg^−1^ soil). Two biochar types MBC and WBC were mixed with the soil at 2% (w/w) as follows:

- un-inoculated plants grown without WBC or MBC biochar,
- un-inoculated plants grown in soil amended with WBC,
- un-inoculated plants grown in soil amended with MBC,
- inoculated plants with *Pseudomonas putida* L2 grown in soil without WBC or MBC chars,
- inoculated plants with *P. putida* L2 grown in soil amended with WBC char,
- inoculated plants with *P. putida* L2 grown in soil amended with MBC char,
- inoculated plants with *Stenotrophomonas pavanii* L8 grown in soil without WBC or MBC chars,
- inoculated plants with *S. pavanii* L8 grown in soil amended with WBC char,
- inoculated plants with *S. pavanii* L8 grown in soil amended with MBC char.

Each of the treatments contained four groups of twenty-four plants and were arranged in a randomized block design. For plant inoculation, bacterial isolates were grown in Tryptic soy broth (TSB) medium for two days, centrifuged, washed in PBS, and re-suspended with a bacterial density of 10^8^ CFU/mL. The seedling roots were dipped into bacterial suspension prior to planting into soil. The plants were grown at a temperature range of 24–28 °C day and 18–20 °C night under greenhouse conditions for 34 days. At harvest, root rot symptoms of washed plants were determined as indicated by browning and lesions.

### 2.7. Plant Growth Experiment with Endophytic Bacteria and Biochar

The plant growth chamber experiments with nine treatments were set up as described above. The lupin seeds were surface-sterilized using 70% ethanol and 10% v/v NaOCl. Afterward, the seeds were washed five times with sterile distilled water and transferred to paper tissue for germination in a dark room at 25 °C for 3–4 days. The bacterial strains *P. putida* L2 and *S. pavanii* L8 were grown in TSB medium for two days, and the culture suspension was re-suspended in PBS with a final bacterial density of 10^8^ CFU mL^−1^. Germinated seeds were dipped into the bacterial suspensions and transferred to pots. Three seeds were sown in each pot, and, after one week, the seedlings were thinned to one plant per pot. Each treatment included four pots and was arranged in a randomized complete block design with three replications (total of 12 plants). Plants were grown for 30 days at a temperature of 24 °C/16 °C (day/night) and humidity of 50–60%. At harvest, the roots and shoots were oven-dried at 70 °C for 48 h, and dry weight was determined.

### 2.8. Survival of Endophytic Bacterial Strains

The rifampicin (200 µg/mL) resistant mutants of the wild-type strain were obtained following the method of Glandorf et al. [45] and were used for the colonization studies. Mutants of *P. putida* L2 and *S. pavanii* L8 that were marked with antibiotic resistance were obtained by plating the parental strain onto TSA agar plates containing increasing concentrations of 50, 100, 150, and 200, µg/mL rifampicin. After incubation, isolates were selected based on similarities in colony morphology and growth rate with the parent strain and were re-cultured on medium containing 200 µg/mL rifampicin to ensure stability of the antibiotic resistance.

Plants were grown in pots as described above. Lupin seedlings were coated with bacteria by dipping the seedlings in bacterial suspensions that resulted in 10^8^ CFU mL^−1^ seeds. Plants were grown for 30 days at a temperature of 24 °C/16 °C (day/night) and at a humidity of 50–60%. At harvest, the adhering soil was removed from plant roots, and 1 g roots was shaken in 9 mL sterile PBS. The resulting suspensions were evaluated for colony forming units (CFUs) according to the dilution-plate method in TSA agar with the addition of 200 µg/mL rifampicin. After incubation for 2–3 days at 28 °C the re-isolated, rifampicin-resistant strains were identified for their colony characteristics [46].

### 2.9. Statistical Analysis

Data were tested for statistical significance using the analysis of variance package included in Microsoft Excel 2010. Comparisons were performed using Student’s t-test. Mean comparisons were conducted using the least significant difference (LSD) test (*P* = 0.05).

## 3. Results

### 3.1. Response of Lupin to the Biochar Types

**Figure 1:**
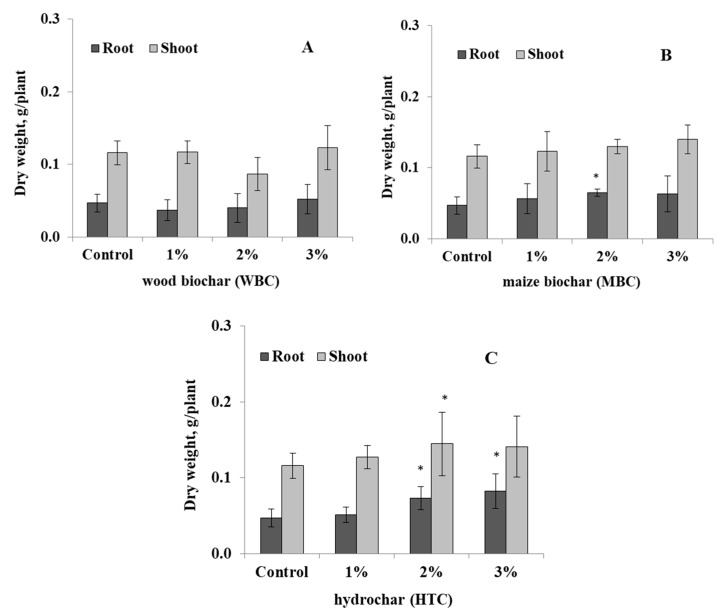
Root and shoot dry weights of lupin grown in a greenhouse for 30 days in a sandy soil containing wood biochar (WBC) (**A**), maize biochar (MBC) (**B**), and HTC-char (**C**) at concentrations of 1%, 2%, and 3% w/v, respectively. Columns represent means for six plants (*N* = 6), with error bars showing the standard deviation.

The response of narrow-leafed lupin to the type of biochar was studied at 1%, 2%, and 3% concentrations in pot experiments. The shoot and root biomass of lupin were not significantly affected by WBC soil amendments at 1–3% (Figure 1A). Correspondingly, the root and shoot growth of lupin under MBC char was not affected, but only root dry weight was increased at 2% MBC amendment (Figure 1B). However, the root dry weight of lupin was significantly increased by 54–75%, and the shoot dry weight was increased by up to 21–25% by HTC char amendment at 2% concentration, respectively (Figure 1C).

**Figure 2:**
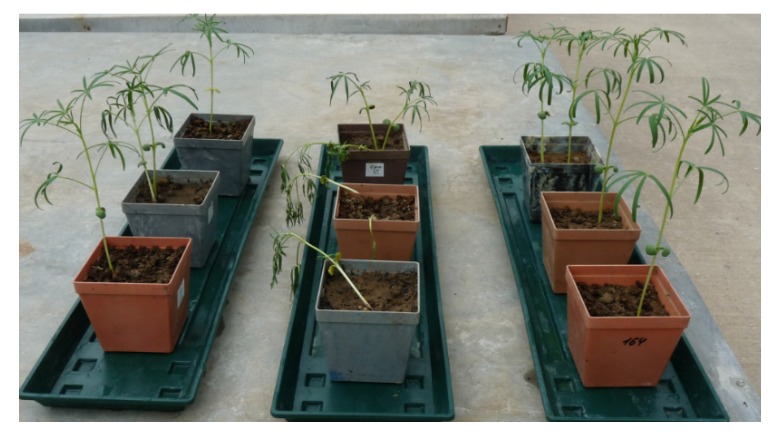
The effect of soil amendments with maize biochar (MBC, left position), wood biochar (WBC, middle position), and HTC-char (HTC, right position) on disease incidence of lupin after 30 days of growth.

The plants grown in soils amended with 2% and 3% w/v WBC and MBC chars showed disease symptoms of 40–50% and 10–20%, respectively (Figure 2). Plants grown in soil without biochar and in soil with HTC char were healthy, and no disease incidence was found.

### 3.2. Fungal and Bacterial Isolates

The selected fungal isolate showed root rot symptoms after plant pathogenicity tests and was identified as *F. solani*. The biological control capability of endophytic bacteria against root rot of lupin caused by *F. solani* was examined.

A total of 25 bacterial isolates were isolated from plant tissues of lupin. The RFLP analysis was conducted to eliminate similar isolates, and as a result, only nine isolates were selected and identified using the BLAST algorithm and compared with related strains from NCBI GenBank (Table 2).

**Figure 3:**
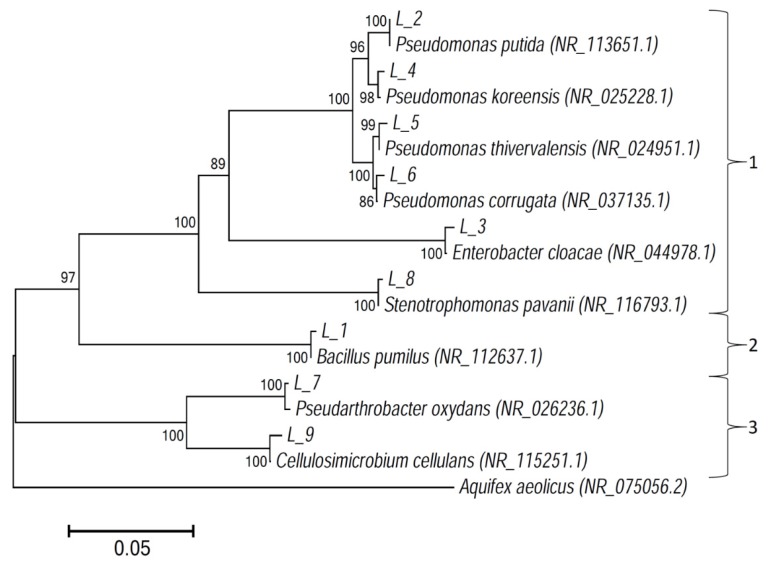
Neighbor-joining phylogenetic tree based on 16S rRNA gene sequences isolated from endophytic bacteria of lupin, showing the relationship of isolated strains to their closest relatives in GenBank. All presented strains were related to three phyla: 1—Proteobacteria, 2—Firmicutes, 3—Actinobacteria.

All strains were identified using BLAST and compared with similar strains from the NCBI GenBank. The 16S rRNA sequences similarities of endophytic bacteria isolated from plant tissue of lupin with sequences from GenBank are shown in Table 2. All strains were identical with a similarity of 98.72–99.25% to those registered in GenBank. The isolates were identified as *Bacillus pumilus* L1, *P. putida* L2, *Enterobacter cloacae* L3, *Pseudomonas koreensis* L4, *Pseudomonas thivervalensis* L5, *Pseudomonas corrugata* L6, *Pseudarthrobacter oxydans* L7, *S. pavanii* L8, and *Cellulosimicrobium cellulans* L9 (Table 2, Figure 3). A phylogenetic tree was constructed showing the closest relatives of the isolates (Figure 3).

All nine endophytes were screened for multiple plant growth-promoting traits. Most of the bacterial isolates exhibited one or more plant growth-promoting activities (Table 3). Among nine isolates only two isolates *P. putida* L2 and *S. pavanii* L8 were able to produce IAA (5.6 and 4.7 µg mL^−1^), were able to produce one or more cell wall degrading enzymes, and were highly effective in vitro against the *F. oxysporum* and *F. solani* (Table 3). HCN and siderophores were produced only by *P. putida* L2.

### 3.3. Biological Control of Lupin Root Rot under Biochar Amended Soil

**Figure 4:**
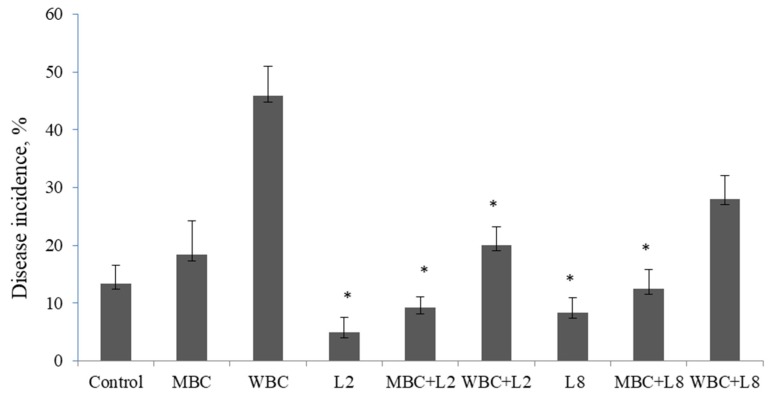
Biological control of lupin root rot caused by *F. solani* using endophytic bacteria *P. putida* L2 and *S. pavanii* L8. The soil samples were infested with *F. solani*, and plants were grown in a greenhouse for 30 days in sandy soil containing either no biochar (Control), maize biochar (MBC), or wood biochar (WBC), with or without endophytic bacteria. Columns represent means for six plants (*N* = 6), with error bars showing the standard deviation.

**Figure 5:**
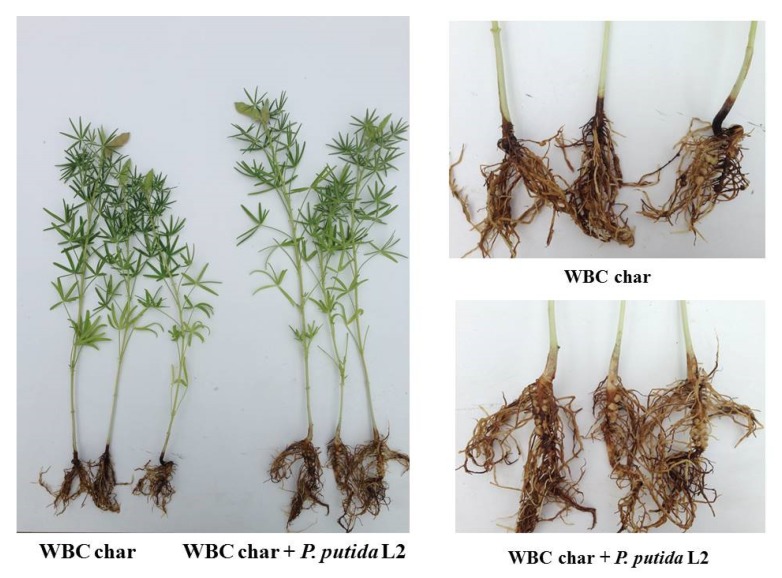
Biological control of lupin root rot caused by *F. solani* by endophytic bacteria *P. putida* L2. Plants were grown in soil amended with WBC char infested with *F. solani.*

The two selected isolates, *P. putida* L2 and *S. pavanii* L8, were chosen to evaluate their capability to suppress lupin root rot caused by *F. solani*. The portion of diseased plants grown in soil infested with the pathogen was 13%. The disease incidence was increased by 18% under MBC amended soil and by 45% under WBC soil amendment (Figure 4). The bacterial isolates, *P. putida* L2 and *S. pavanii* L8, demonstrated a disease reduction up to 9–12% under MBC amended soil compared to control plants without biochar (13%) and un-inoculated plants grown under MBC (18%) (Figure 5). The number of pathogen-infected plants was reduced up to 20% and 28% by bacterial isolates *P. putida* L2 and *S. pavanii* L8 under WBC soil amendment, whereas un-inoculated plants grown under WBC char showed a disease incidence up to 45%.

### 3.4. Plant Growth, Nodulation, and Bacterial Survival in the Root System

**Figure 6:**
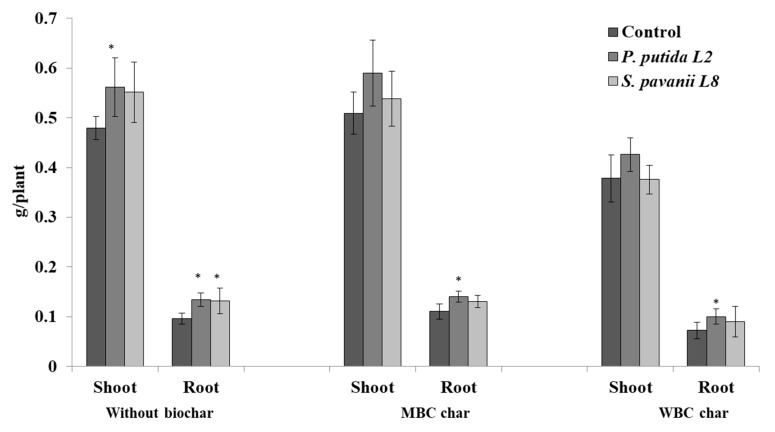
The effect of endophytic bacteria *P. putida* L2 and *S. pavanii* L8 on shoot and root growth of lupin. Plants were grown in a greenhouse for 30 days in sandy soil containing either no biochar (Control), maize biochar (MBC), or wood biochar (WBC). Columns represent means for six plants (*N* = 6), with error bars showing the standard deviation.

The endophytic bacteria that showed biocontrol ability to control the root rot of lupin were evaluated for their plant growth-promoting activities under the amendment of two different biochar types. The responses of lupin to the two biochar types were different, being higher in soil amended with MBC compared to those in soil amended with WBC (Figure 6). The shoot and root biomass of un-inoculated lupin grown under MBC char were increased by 6% and 14% compared to control plants, respectively. Bacterial inoculants *P. putida* L2 and *S. pavanii* L8 increased lupin root and shoot growth in soil without biochar amendment by 17–39% and 15–37%, respectively (Figure 6). However, there were no significant effects on the shoot growth of inoculated plants grown in soil amended with MBC and WBC chars. The root biomass of lupin was increased by inoculation of plants with *P. putida* L2 in MBC and WBC amended soil (Figure 6). Compared with plants grown in soil amended with MBC and WBC chars, the weight of shoots and roots was not affected by bacterial inoculation with *S. pavanii* L8.

**Figure 7:**
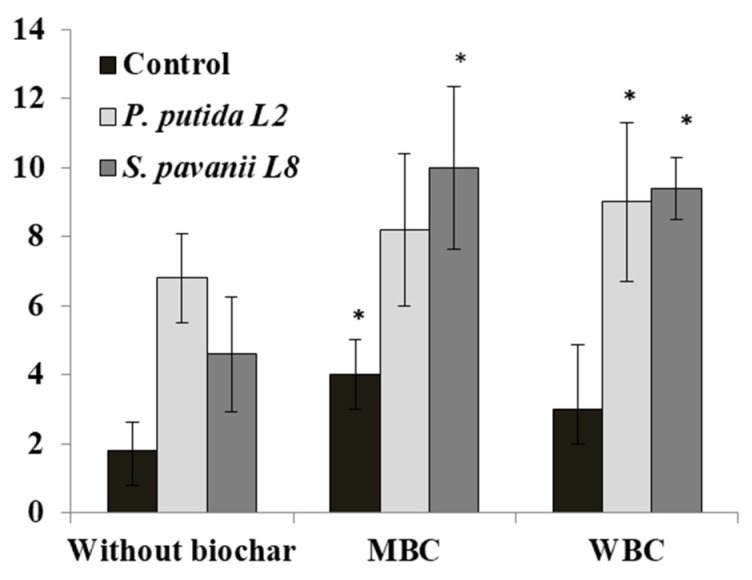
The effect of endophytic bacteria *P. putida* L2 and *S. pavanii* L8 on the nodule number of lupin. Plants were grown in a greenhouse for 30 days in sandy soil containing either no biochar (Control), maize biochar (MBC), or wood biochar (WBC). Columns represent means for six plants (*N* = 6), with error bars showing the standard deviation.

Both endophytic bacteria *P. putida* (L2) and *S. pavanii* (L8) improved the symbiotic performance of lupin with its rhizobia under soil amended with MBC and WBC chars. The nodule numbers of lupin inoculated with *P. putida* L2 and *S. pavanii* L8 were 6.8 and 4.6 grown in soil without biochar, while under WBC and MBC biochar amendment nodule numbers increased to 9.0 and 10.0 per plant, respectively (Figure 7).

Rifampicin-resistant mutants of bacterial isolates *P. putida* L2 and *S. pavanii* L8 were tested for their survival in the root of lupin grown without and with biochar amendment. The results showed that both bacterial isolates were able to survive in the rhizosphere of lupin. The root colonization in the rhizosphere of lupin was 2.1 × 10^3^ ± 1.4 with *P. putida* L2, and 3.2 × 10^3^ ± 1.9 with *S. pavanii* L8 in soil without biochar amendment. The CFUs of bacterial isolates were slightly higher in WBC and MBC amended soils, being 3.2 × 10^3^ ± 0.7 and 4.2 × 10^3^ ± 1.2 (CFU/g of fresh root) with *P. putida* L2 and 3.9 × 10^3^ ± 1.6 and 3.8 (CFU/g of fresh root) with *S. pavanii* L8, respectively.

## 4. Discussion

Biochar amendments have been reported to elicit complex responses in plants [4,6,15], including plant growth stimulation or suppression of several soil-borne diseases [47,48]. However, the beneficial effect depends on the type of biochar feedstock, the technique of preparation (e.g., pyrolysis or carbonization), and finally, concentration [15,16]. Overall, our experiment showed that the shoot and root biomass of lupin responded differently to types of biochar and biochar concentrations, i.e., MBC char produced more benefits to lupin growth as compared to WBC char. Reibe et al. [33] reported similar observation for MBC char amendments increasing dry biomass of wheat as compared to WBC char or plants grown without any biochar application. Joseph et al. [49] reported that the interaction of biochar with environmental conditions is another crucial requirement for revealing contrasting effects, which might depend on the physicochemical properties of biochars. For example, Butnan et al. [25] observed reduced plant growth on a sandy Ultisol amended with eucalyptus wood-derived biochar produced by pyrolysis (800 °C), whereas biochar produced at a lower temperature (350 °C) provided higher benefits. Shen et al. [50] reported that the addition of biochar from willow woodchips increased plant growth of *Lotus pedunculatus* cv barsille, whereas pine-based biochar did not show any positive effect on plant growth.

There are also numerous reports on the positive effect of biochar in reducing plant pathogens [51], e.g., *R. solani* of cucumber [16] or *F. oxysporum* f. sp. *lycopersici* of tomato [14]. However, some biochar types showed an increased disease incidence by providing favorable conditions for pathogenic fungi, e.g., 3% green waste biochar increased the disease incidence of tomato wilt caused by *F. oxysporum* f. sp. *lycopersici* [14]. Correspondingly, we observed an increased disease incidence of lupin wilt caused by *F. solani* under WBC char, whereas no diseased plants were observed in control plots. However, the mechanisms of biochar to suppress or induce soil-borne diseases were not sufficiently revealed because they depend upon several factors, including chemical and physical characteristics of biochar [16] as well as soil conditions and properties. In earlier studies, Sanogo and Yang [52] reported that the development and growth of *Fusarium* species was faster at higher soil pH 8.2 compared to that at lower soil pH 5.7.

The capability of endophytic bacteria living within plant tissues to inhibit plant pathogens was reported in numerous studies [22,53,54]. Silva et al. [55] observed a biological control of coffee leaf rust caused by *Hemileia vastatrix* with endophytic bacteria such as *Bacillus megaterium* and *Microbacterium testaceum.* In another study, *Bacillus cereus* decreased wilt symptoms of tomato caused by *F. oxysporum* [56]. In our study, endophytic bacteria *P. putida* L2 and *S. pavanii* L8 were able to reduce lupin root rot caused by *F. solani* in WMB and MBC amended soil. In a previous study, the endophytic *Bacillus subtilis* NUU4 with antagonistic activity against *F. oxysporum*, *F. solani*, and *F. culmorum* reduced disease incidence of chickpea root rot [54]. The plant inoculation with *P. putida* L2 and *S. pavanii* L8 increased lupin root and shoot biomass grown in soil without biochar. The root biomass of lupin was significantly increased by *P. putida* L2 under both MBC and WBC char. Lupin treated with *Bradyrhizobium* sp. and growing in soil amended with biochar had higher root and shoot biomass as compared to un-inoculated control plants or direct inoculation with *Bradyrhizobium* sp., alone [9]. Correspondingly, soil amendment with biochar from citrus wood provided favorable conditions for bacterial proliferation under sweet pepper (*Capsicum annuum* L.) [18]. Pietikäinen et al. [57] reported that the survival of bacteria, which were adsorbed to biochar surfaces, was due to biochar properties protecting bacteria in the soil. In another study, the number of rhizobial cells in soil under soybean were increased by the addition of biochar mixed with compost [58]. Hale et al. [59] observed that pinewood biochar based inoculum of *E. cloacae* UW5 resulted in a significant increase in cucumber root branching and total root length giving evidence for the role of biochar in effects on root architecture. The ability of endophytic bacteria colonizing the internal part of plants to stimulate the root system by better nutrient uptake was reported in numerous studies [53,54,60,61]. The colonization of inoculated bacterial isolates in the root system is an important trait, especially in a highly competitive environment [62,63]. Moreover, it is the very first step in beneficial interactions of the plant with a microbe. The inoculated endophytic bacteria were able to survive in the rhizosphere of lupin grown in soil without biochar and biochar amendment for 30 days. It has been previously reported that improved plant–microbe interactions resulted from biochar effects on soil microbial diversity and activity [64], e.g., biochar maintains bacterial proliferation residing inside pores by supplying nutrients and improving aeration [65].

Several mechanisms by which endophytic bacteria enhance plant growth, improve stress tolerance, and protect plants from various diseases were described. These include the production of phytohormones such as indole 3-acetic acid [9,54], antifungal metabolites [62,66], and cell wall degrading enzymes such as cellulase or chitinase [67]. In our previous work, the endophytic strain *B. subtilis* NUU4 that reduced root rot incidence in chickpea and stimulated plant growth demonstrated an antagonistic activity against *F. oxysporum*, *F. solani*, and *F. culmorum* and produced IAA, as well as cell wall degrading enzymes. Similar observations were found by Abdallah et al. [56], where the wilt symptoms of tomato caused by *F. oxysporum* were decreased by *B. cereus* antagonistic against *F. oxysporum* f. sp. *lycopersici*. The endophytic bacteria that synthesized IAA stimulated the root system architecture and also increased root hairs, which resulted in better absorption of nutrients [68].

## 5. Conclusions

The results presented in this study showed that soil amended with 2% MBC and HTC improved root and shoot growth of lupin. However, the disease symptoms of lupin root rot occurred in soil amended with WBC char at a rate of 2% and 3%. Plants under HTC char were healthy, and no disease incidence was found. The endophytic bacteria *P. putida* L2 and *S. pavanii* L8 isolated from lupin were able to reduce root rot caused by *F. solani* under WBC amended soil. Furthermore, the lupin growth was also increased by inoculation with endophytic bacteria in MBC and WBC amended soil treatments. The soil amended with MBC and WBC supported better survival of inoculated bacteria in the rhizosphere of lupin. Future research is required to reveal the mechanisms of biochar-induced disease incidence in more detail, as well as the traits endophytic bacteria use to control lupin root rot caused by F. *solani* in soil amended with biochar, especially considering soil physicochemical properties.

## Figures and Tables

**Table 1:** Characterization of chars [33].

Material	DM (%FM)	Ash (%DM)	Ct (%DM)	Nt (%DM)	P (g/kg FM)	K (g/kg FM)	pH	EC
HTC-char	47.39	3.19	64.55	2.09	1.02	3.58	5.25	0.30
MBC	92.85	18.42	75.16	1.65	5.26	31.12	9.89	3.08
WBC	55.09	16.64	77.62	0.72	1.24	7.8	9.35	1.71

FM, fresh matter; DM, dry matter; MBC, maize biochar; HTC, hydrochar; WBC, wood biochar; EC, electrical conductivity.

**Table 2:** Sequence similarities of endophytic bacteria isolated from the roots of lupin grown under field conditions with sequences registered in GenBank.

Sequences of Isolated Strains Deposited to GenBank			Closest Match among Bacteria (16S rRNA Genes) (GenBank)		
Strain	Length (bp)	Accession Number	Reference Strains	Accession Number	Percent Identity
L_1	1456	MH636785	*Bacillus pumilus*	NR_112637.1	99.25
L_2	1437	MH636786	*Pseudomonas putida*	NR_113651.1	99.24
L_3	1404	MH636787	*Enterobacter cloacae*	NR_044978.1	98.72
L_4	1409	MH636788	*Pseudomonas koreensis*	NR_025228.1	99.15
L_5	1406	MH636789	*Pseudomonas thivervalensis*	NR_024951.1	99.22
L_6	1445	MH636790	*Pseudomonas corrugata*	NR_037135.1	99.03
L_7	1436	MH636791	*Pseudarthrobacter oxydans*	NR_026236.1	99.23
L_8	1485	MH636792	*Stenotrophomonas pavanii*	NR_116793.1	99.06
L_9	1358	MH636793	*Cellulosimicrobium cellulans*	NR_115251.1	98.75

**Table 3:** Characterization of endophytic bacterial isolates.

Bacterial Isolates				Exo-Enzymes			Antagonistic Activity				
	HCN production	Siderophore	IAA (µg/mL)	Protease	Cellulase	Chitinase	*F.oxysporum*	*F.solani*	*F.culmorum*	*A.alternata*	*B.cinerea*
*B. pumilus* L1	−	−	−	−	−	−	−	−	−	−	−
*P. putida* L2	+	+	+	−	+	+	+	+	+	−	−
*E. cloacae* L3	−	−	−	−	−	−	−	−	−	−	−
*P. koreensis* L4	−	−	−	−	+	−	+	+	−	−	−
*P. thivervalensis* L5	−	−	−	−	−	+	−	−	−	−	−
*P. corrugata* L6	−	−	−	+	−	−	−	−	−	+	+
*P. oxydans* L7	+	−	−	−	−	−	−	−	−	−	−
*S. pavanii* L8	−	−	+	−	+	+	+	+	−	−	+
*C. cellulans* L9	−	−	+	+	−	−	−	−	−	−	−

“+” positive; “−“ negative”.
